# Insomnia in chronic obstructive pulmonary disease and associations with healthcare utilization and costs

**DOI:** 10.1186/s12931-023-02401-w

**Published:** 2023-03-25

**Authors:** Faith S. Luyster, Monique Y. Boudreaux-Kelly, Jessica M. Bon

**Affiliations:** 1 School of Nursing, University of Pittsburgh, 3500 Victoria St, 415 Victoria Building, Pittsburgh, PA 15241 USA; 2 VA Pittsburgh Healthcare System, Pittsburgh, PA USA; 3 Division of Pulmonary, Allergy, and Critical Care Medicine, University of Pittsburgh, School of Medicine, Pittsburgh, PA USA

**Keywords:** Sleep initiation and maintenance Disorders, Pulmonary Disease, Chronic Obstructive

## Abstract

Insomnia has been linked to adverse chronic obstructive pulmonary disease (COPD) outcomes including exacerbations, yet its impact on COPD-related healthcare utilization and costs is unknown. In this study, we investigated the associations between insomnia and healthcare utilization and costs in patients with COPD. A retrospective cohort of veterans with COPD were identified from national Veterans Affairs administration data for fiscal years 2012–2017. Insomnia was operationalized as having an insomnia diagnosis based on International Classification of Disease codes or having a prescription of > 30 doses of a sedative-hypnotic medication in a given fiscal year. The index date for insomnia was the first date when dual criteria for COPD and insomnia was met. The index date for those without insomnia was set as the COPD index date. Our primary outcomes were 1-year healthcare utilization and costs related to outpatient visits and hospitalizations after index date. COPD-related healthcare utilization variables included number of prescription fills of corticosteroids and/or antibiotics and outpatient visits and hospitalizations with a primary diagnosis of COPD. Out of 1,011,646 patients (96% men, mean age 68.4 years) diagnosed with COPD, 407,363 (38.8%) had insomnia. After adjustment for confounders, insomnia was associated with higher rates of outpatient visits, hospitalizations, and fills for corticosteroids and/or antibiotics, longer hospital length of stay, and $10,344 higher hospitalization costs in the 12 months after index date. These findings highlight the importance of insomnia as a potentially modifiable target for reducing the burden of COPD on patients and healthcare systems.

## Introduction

Chronic obstructive pulmonary disease (COPD) is a highly prevalent condition, with 6.2% of adults reporting being diagnosed with COPD in 2017, and its burden is anticipated to increase as the population continues to age [1, 2]. Acute exacerbations of COPD lead to deterioration in lung function and quality of life, increased risk for mortality, and frequently require emergency department (ED) visits and hospitalization, therefore contributing to excess healthcare use [3–5]. Exacerbations along with complex care needed to address multiple comorbidities commonly present among patients with COPD imparts a great economic burden to healthcare systems [6]. Identifying modifiable risk factors is critical for the prevention of COPD exacerbations and the consequent reduction in healthcare utilization and costs.

Insomnia is a common complaint among patients with COPD [7–10]. Sleep difficulties in COPD may plausibly arise from smoking, psychiatric and medical comorbidities including depression, anxiety, obstructive sleep apnea, restless legs syndrome, and pain, supplemental oxygen use and medications for the treatment of COPD, and nocturnal awakenings due to nighttime respiratory symptoms such as cough and dyspnea [11]. Irrespective of etiology, insomnia has been linked to adverse outcomes in COPD including reductions in quality of life and daytime function, COPD-related symptoms and incident exacerbations, and increased risk for mortality [12–15].

Untreated insomnia is associated with substantial healthcare utilization and costs, particularly among older adults and those with comorbidities [16–22]. Relative to individuals without insomnia, rates of inpatient, ED, and outpatient care and healthcare costs, primarily driven by inpatient costs, are significantly higher among individuals with insomnia [18, 20]. Even after controlling for comorbidities, individuals with insomnia continue to demonstrate greater healthcare utilization and costs [16, 20]. When coupled with comorbidities, healthcare costs are as much as 80% higher in the 12 months after insomnia diagnosis [16]. To our knowledge, no prior studies have examined the impact of insomnia on healthcare utilization and costs in patients with COPD.

This study investigated the associations between insomnia and COPD-related healthcare utilization and costs utilizing a large cohort of patients with COPD receiving care within the national Veterans Health Administration (VHA). We hypothesized that patients with COPD and insomnia would have greater rates of COPD-related healthcare utilization including outpatient visits, hospitalizations, and number of prescription fills of corticosteroids and/or antibiotics and higher COPD-specific outpatient visit and hospitalization costs compared to those with COPD only.

## Methods

### Data source and study cohort

This a retrospective cohort of veterans with COPD who utilized health services through the VHA system between fiscal years (FY) 2012 and 2017. The Veteran Affairs (VA) Corporate Data Warehouse was utilized to access nationwide electronic medical record data on demographics, diagnoses, prescription fills, and encounters. Patients were classified as having COPD if they had (1) at least two outpatient encounters or (2) at least one hospitalization with a primary diagnosis of COPD based on International Classification of Diseases, Ninth Revision, Clinical Modification (ICD-9-CM) codes or Tenth Revision, Clinical Modification (ICD-10-CM) codes [23, 24]. This approach has been found to reduce misclassification and has been utilized in previous studies [25]. The date of the first hospitalization for COPD or the date when 2 outpatient encounters occurred was set as the COPD index date. The VA Pittsburgh Healthcare System Institutional Review Board approved this study (IRB# PRO00002714).

### Exposure of interest

From this population of patients with COPD, we identified two populations: those with insomnia and those without insomnia. Patients were classified as having insomnia if they met the following criteria: (1) insomnia diagnosis on at least one occasion between FY2012 and FY2017 based on ICD-9-CM and ICD-10-CM codes utilized in a prior study of veterans [26] or (2) had a prescription fill for > 30 doses in a given fiscal year of at least one of the following sedative-hypnotic medications: zolpidem, zaleplon, eszopiclone, temazepam, triazolam, ramelteon, trazodone, amitriptyline and doxepin (including only doses < 100 mg per day of the last three medications). The insomnia medications were selected based on clinical practice guidelines [27], on- and off-label use of medications for insomnia treatment, and everyday clinical practice. We chose to identify insomnia by either having a diagnosis or prescription fills for sedative-hypnotic medications as recent studies suggest that insomnia is underdiagnosed in VA electronic health records and that sedative-hypnotics are commonly prescribed in the absence of a formal insomnia diagnosis [26, 28, 29]. The index date for those with insomnia was the first date when dual criteria for COPD and insomnia was met. The index date for those without insomnia was set as the COPD index date.

### Outcomes of interest

The outcomes of interest were 1-year healthcare utilization and costs after the index date. COPD-related healthcare utilization variables included the number of outpatient visits (including outpatient clinic visits and emergency department visits) and hospitalizations with a primary diagnosis was COPD. Length of hospital stay was also captured. Pharmacy utilization was determined by a prescription fill of antibiotics and/or corticosteroids. Costs related to COPD-specific outpatient visits (specifically for outpatient clinic visits) and hospitalizations were obtained from cost estimates from Health Economics Resource Center average cost data files, which are modeled from Medicare claims data and adjusted to reflect total annual VHA expenditures [30, 31]. Only VA medical costs data were included, thus, costs incurred from VA pharmacy, Medicare Advantage, Medicaid, care paid for by VA but received in the community, and private insurance (community care) were excluded. Inpatient cost estimates were based on the national average cost of a hospital stay given its Diagnosis Related Group (DRG), overall length of stay, and days in intensive care [30]. Outpatient cost estimates were based on estimates provide by Medicare using Current Procedure and Terminology Codes assigned to the VA visit [31].

### Covariates

Based on prior work examining healthcare utilization and costs within the VA, we controlled for potential confounders including sociodemographic and clinical factors and VA-specific variables [32–34]. Sociodemographic factors included age at index date, sex, race and ethnicity (non-Hispanic White, Non-Hispanic Black, Hispanic, or other), and marital status (married, never married, widowed/divorced/separated, or other). Clinical factors included smoking status (current vs. former or never), body mass index (BMI) expressed as kg/m^2^, and psychiatric and medical diagnoses identified by ICD-9-CM or ICD-10-CM codes. VA-specific variables included combat deployment and receipt of VA service-connected disability compensation (none, < 50%, ≥ 50%), which is given to veterans based on conditions determined to be associated with military service.

### Statistical analysis

Descriptive bivariate statistics were used to compare patients with COPD with and without insomnia. Continuous variables were analyzed using Student’s t-test and categorical variables were analyzed using Chi-square test. Wilcoxon rank-sum tests assessed unadjusted differences in healthcare utilization between groups. Effect sizes of the bivariate differences between patients with and without insomnia were measured by Cramer’s V [35] for categorical data and Cohen’s d [36] for continuous variables. Strength of effect size was determined by established interpretive values for each test, as described in Table 1. The effect of insomnia on COPD-related healthcare utilization was examined using multivariate negative binomial regressions and were indicated as incident rate ratios. Quantile regressions were used to examine the effect of insomnia on COPD-related outpatient and hospitalization costs among patients who incurred costs and were indicated as differences in quantile costs. Quantile regression is often used to model costs because it allows the same modeling complexity found in linear regression while being robust against outliers. Similar to linear regression, it uses estimates to compare conditional values of the costs across values of the predictor, in this case insomnia. However, instead of utilizing the method of least squares to estimate cost values, it uses non-parametric quantiles across values of the predictor while adjusting for covariates. All multivariate analyses were adjusted for age, sex, race, marital status, smoking status, service connection, body mass index, and individual comorbid conditions (obstructive sleep apnea, gastroesophageal reflux disease, restless legs syndrome, asthma, diabetes, ischemic heart disease, stroke, human immunodeficiency virus, depression, anxiety, and post-traumatic stress disorder). Statistical significance was set at P < 0.05. Statistical analyses were conducted with SAS version 9.4 (SAS Institute, Cary, NC).

**Table 1:** Demographic and baseline clinical characteristics by insomnia status

Characteristic	Insomnia (n = 407,969)	No Insomnia (n = 603,677)	Effect Size^a^
Age (years), mean (SD)	65.6 (10.7)	70.3 (10.9)	0.44
Sex, male, *n (%)*	387,395 (94.9)	586,246 (97.1)	0.06
Race/Ethnicity, *n (%)*			0.06
White	308,410 (75.6)	462,047 (76.5)	
Black	52,229 (12.8)	63,836 (10.6)	
Hispanic	13,917 (3.4)	14,855 (2.5)	
Other/Missing	33,413 (8.2)	62,939 (10.4)	
Marital Status, *n (%)*			0.06
Married	203,047 (49.7)	313,258 (51.9)	
Never married	167,807 (41.1)	217,098 (35.9)	
Widowed/Divorced/Separated	36,455 (8.9)	710,264 (11.8)	
Other/Missing	660 (0.2)	2,057 (0.3)	
Current smoker, *n (%)*	207,076 (50.8)	247,855 (41.1)	0.10
Combat deployment, *n (%)*	43,381 (10.6)	56,057 (9.3)	0.02
Any Service connection, *n (%)*	246,566 (60.4)	266,034 (44.1)	0.16
VA service connection compensation, *n (%)*			0.19
None	149,069 (36.5)	317,967 (52.7)	
< 50%	65,861 (16.1)	110,935 (18.4)	
≥ 50%	180,761 (44.3)	155,254 (25.7)	
Missing	12,278 (3.0)	19,521 (3.2)	
BMI (kg/m^2^), mean (SD)	30.0 (7.1)	27.7 (6.2)	0.31
Any Comorbidity, *n (%)*	378,945 (92.9)	464,837 (77.0)	0.21
Select Comorbidities, *n (%)*			
Obstructive sleep apnea	161,661 (39.6)	45,371 (7.5)	0.38
Gastroesophageal reflux disease	201,840 (49.5)	213,591 (35.4)	0.14
Restless legs syndrome	20,337 (4.9)	10,312 (1.7)	0.09
Asthma	31,513 (7.7)	29,642 (4.9)	0.06
Diabetes	184,343 (38.5)	205,013 (33.9)	0.11
Ischemic heart disease	184,852 (45.3)	240,723 (39.9)	0.05
Stroke	19,963 (4.9)	21,285 (3.5)	0.03
Human immunodeficiency virus	22,577 (5.5)	23,829 (3.9)	0.03
Depression	87,512 (21.5)	32,631 (5.4)	0.24
Anxiety	39,131 (9.6)	18,190 (3.0)	0.14
Post-traumatic stress disorder	129,897 (31.84)	62,753 (10.4)	0.26

Definition of abbreviations: BMI = body mass index; SD = standard deviation, VA = Veterans Affairs

^a^ By Cramer’s V for categorical data and Cohen’s d for continuous data, as appropriate. Cramer’s V: >0.05 = weak, > 0.10 = moderate, > 0.15 = strong. Cohen’s d: 0.20 = small, 0.50 = medium, 0.80 = large

## Results

### Participant characteristics

Our analysis included 1,011,646 patients who were diagnosed with COPD between FY2012 and FY2017. The cohort was primarily non-Hispanic White (76.8%) and male (96.2%) with a mean age of 68.4 ± 11.1 years. Of those with COPD, 407,969 (38.8%) were identified as having insomnia. Table 1 summarizes sample demographics according to the presence of insomnia. Patients with insomnia were younger, more likely to be female, racial/ethnic minorities, current smokers, never married, previously deployed, and receiving greater service connection. The occurrences of all comorbidities, especially obstructive sleep apnea, depression, anxiety, post-traumatic stress disorder, and restless legs syndrome, in patients with insomnia were higher than patients without insomnia. BMI was also higher in those with insomnia. Effect sizes indicated strong associations between insomnia and presence of service connection and any comorbidity, obstructive sleep apnea, depression, and post-traumatic stress disorder (Table 1). Younger age was moderately associated with insomnia.

### Association between insomnia and 1-year COPD-related healthcare utilization and costs after index date

Table 2 shows the relationship between insomnia and COPD-related healthcare utilization and costs in the year after index date. In unadjusted analyses, patients with COPD and insomnia had more total COPD-related outpatient visits (4.81 versus 3.78, P < 0.001), hospitalizations (1.57 vs. 1.46, P < 0.001), longer hospitalization length of stay (90.70 vs. 65.51, P < 0.001), and prescription fills for corticosteroids and/or antibiotics (4.80 vs. 3.64, P < 0.001) over the 1-year. Compared to those without insomnia, patients with insomnia had greater 1-year outpatient ($885 vs. $806, P < 0.001) and hospitalization ($79,428 vs. $69,068, P < 0.001) costs. Multivariable adjusted utilization and cost rate ratios for patients with COPD by insomnia status are also shown in Table 2. After adjustment for covariates, patients with COPD and insomnia had more 1-year outpatient visits and hospitalizations, longer hospitalization length of stay, and prescription fills for corticosteroids and/or antibiotics. These increases in hospitalizations were associated with increases in related costs. No differences in outpatient costs were found between those with and without insomnia after adjusting for covariates.

**Table 2:** Association of insomnia with COPD-related healthcare utilization and costs in 12 months after index date

			Unadjusted analysis		Adjusted analysis	
	Insomnia (n = 407,969)	No Insomnia (n = 603,677)	Difference^a^	95% CI	IRR^b^	95% CI
Healthcare use, mean ± SD						
Outpatient visits	4.81 ± 8.64	3.78 ± 6.22	1.03**	1.00–1.06	1.17**	1.16–1.18
Hospitalizations	1.57 ± 1.15	1.46 ± 0.99	0.11**	0.10–0.12	1.02*	1.02–1.03
Hospitalization LOS (Days)^c^	90.70 ± 114.60	65.51 ± 93.40	25.19**	22.67–27.71	1.26**	1.22–1.31
Prescription fills for steroids and/or antibiotics	4.80 ± 6.08	3.64 ± 4.90	1.16**	1.05–1.26	1.15**	1.13–1.18
**Healthcare costs, median ± IQR**					**Difference at Median** ^**d**^	**95% CI**
Outpatient visit-related costs ($)	885.13 ± 1,276.18	806.54 ± 1,015.48	78.59**	75.74–81.44	3.46	-1.43–8.34
Hospitalization-related costs ($)	79,428.00 ± 209,460.00	69,068.00 ± 159,719.00	10,360.00**	6,936.54–13,783.00	10,344.51**	6,715.18–13,973.84

Definition of abbreviations: CI = confidence interval; COPD = chronic obstructive pulmonary disease; IRR = incidence risk ratio; LOS = length of stay

^a^ Calculated as the difference between patients with insomnia and patients without insomnia. Significance determined by Wilcoxon rank-sum test

^b^ Negative binomial regression model adjusted for age, sex, race, marital status, current smoker, service connection, body mass index, and comorbid conditions

^c^ Maximum 12-month inpatient length of stay set to 365 days

^d^ Quantile regression model adjusted for age, sex, race, marital status, current smoker, service connection, body mass index, and comorbid conditions. Wilcoxon rank test of differences in cost across percentile coefficients for insomnia

*p < 0.05, **p < 0.001

**Fig. 1:**
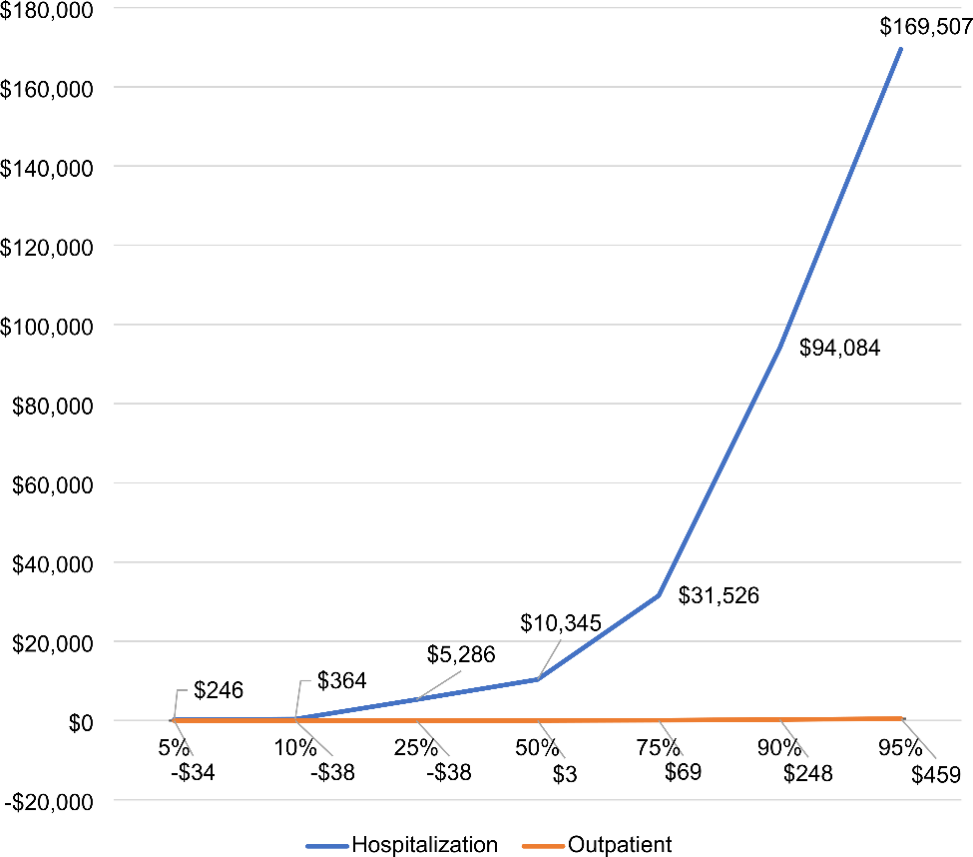
Adjusted differences in total 12-month costs per percentile among patients with insomnia relative to patients without insomnia

Figure 1 shows the differences between insomnia groups for outpatient and inpatient costs at selected percentiles from 5 to 95%. Adjusted insomnia group differences between total 12-month COPD-related costs at the median (50%), 5%, 10%, 25%, 75%, 90%, and 95% percentiles showed that inpatient costs for patients with insomnia were greater than costs for patients without insomnia. At the median, patients with insomnia cost $10,345 more in inpatient care settings than patients without insomnia. Moving across the percentiles from 5 to 95%, the inpatient costs of patients with insomnia ranged from $246 at 5% to $169,507 at 95%. For outpatient costs, adjusted insomnia group differences at the median (50%), 75%, 90%, and 95% percentiles showed that outpatient costs for patients with insomnia were greater than costs for patients without insomnia, but outpatient costs were less for insomnia patients at the 5%, 10%, and 25% percentiles. At the median, patients with insomnia cost $3 more in inpatient care settings than patients without insomnia. Moving across the percentiles from 5 to 95%, the outpatient costs of patients with insomnia ranged from -$34 at 5% to $459 at 95%.

## Discussion

To the best of our knowledge, this study presents for the first time the prevalence of insomnia and its associated impact on utilization of healthcare services and associated costs in a large national cohort with COPD. Results revealed an approximately 4-fold higher prevalence of insomnia in patients with COPD compared to rates reported in the general population [37]. Insomnia was associated with increased COPD-related healthcare utilization and costs. Patients with insomnia had hospital stays that were 38% longer than patients without insomnia, which likely contributed to greater hospitalization-related costs among patient with insomnia.

Prior studies of COPD patients reported the prevalence of insomnia disorder to be between 25% and 47.2% [7, 10, 38] and utilized generally small sample sizes and non-diagnostic insomnia criteria such as questionnaires or specific scales. In contrast, our cohort included over one million patients with COPD and utilized ICD codes and sedative-hypnotic prescription as indicators of insomnia. The high prevalence of insomnia (37%) found in our study aligns with prevalence rates in previous reports that utilized diagnostic insomnia criteria [39, 40]. Age, sex, and co-existing medical and psychiatric conditions have been identified as risk factors for insomnia [41]. In our study and prior studies in patients with COPD, younger age, female sex, current smoking, and physical and mental disorders were found to be associated with insomnia [7, 10]. Of particular note, obstructive sleep apnea, depression, and post-traumatic stress disorder were approximately 3 to 4-times more likely in those with insomnia compared to those without insomnia in our COPD cohort which was comprised of Veterans suggesting a potential critical role of comorbid sleep and psychiatric disorders in the manifestation of insomnia.

Comorbidities among patients with COPD increase the rates of all-cause and COPD-related hospitalizations, length of stay, and in-hospital costs [42–46]. Our study builds upon the current literature by investigating the impact of comorbid insomnia on COPD-related healthcare utilization and costs, showing that insomnia is longitudinally predictive of higher rates of outpatient visits and hospitalizations, longer hospital length of stay, and hospital-related costs even after controlling for other comorbidities. Our results confirm an earlier report demonstrating baseline sleep disturbance suggestive of insomnia as a predictor of COPD-related emergency utilization (hospitalizations or ED visits) over the ensuing year [12]. These findings suggest that insomnia is a strong contributor of healthcare outcomes and costs in patients with COPD. The connection between insomnia and healthcare use and costs may be partially explained by evidence indicating that sleep difficulties independently predict incident COPD exacerbations [47, 48]. In our study, we examined the number of prescription fills for steroids and/or antibiotics, which can be indicative of a COPD exacerbation. Insomnia was longitudinally associated with greater prescription fills for corticosteroids and/or antibiotics, thus providing further support for insomnia being a risk factor for worse COPD outcomes.

There are several potential explanations for the association between insomnia and greater healthcare usage. Sleep disturbance is associated with an elevated systemic inflammatory response, increased C-reactive protein and interleukin-6, which could instigate COPD exacerbations due to increased systemic and airway inflammation [49]. Sleep insufficiency compromises immune function, thus increasing susceptibility to upper and lower respiratory tract infections that can trigger COPD exacerbations [50, 51]. Impairments in memory and attention is common in insomnia and cognitive dysfunction can lead to poor adherence to COPD medications and improper inhaler use [52, 53]. Conversely, insomnia could be an indicator of more severe disease, as increased and unstable COPD symptoms and use of medications such as β-agonists and corticosteroids could lead to sleep disturbances [11]. In addition, insomnia frequently co-occurs with common comorbidities in COPD, such as depression, anxiety, and obstructive sleep apnea, which have been associated with increased risk of COPD exacerbations [54–57]. The relationship between insomnia and COPD is complex and likely multiple mechanisms are in effect at once. Management of multimorbidity in patients with COPD involves identification and treatment of comorbidities [58]. Although treatment of COPD and other comorbid conditions may resolve insomnia symptoms, in some cases insomnia may become self-sustaining and consequently an independent disease process which requires targeted treatment. Cognitive-behavioral therapy for insomnia (CBT-I) is the first-line treatment for insomnia and is associated with reduced healthcare utilization and costs [59, 60]. A recent clinical trial of CBT-I in patients with COPD reported decreases in insomnia and improvements in fatigue and dyspnea [61]. Hypnotics, including benzodiazepine receptor agonists and sedating antidepressants, should be used with caution in patients with severe COPD and used short-term or intermittently in more stable patients as long-term use may be associated with adverse respiratory outcomes [47]. Despite these risks, benzodiazepine receptor agonists are frequently prescribed among patients with COPD [62, 63]. Non-benzodiazepine receptor agonists may have fewer respiratory depressant effects in COPD patients than benzodiazepine receptor agonists [64]. Future studies are needed to examine whether treatment-related reductions in insomnia via CBT-I or pharmacological treatments are associated with reduced healthcare usage and costs in patients with COPD.

Strengths of the study include a large sample size inclusive of all COPD users of the largest integrated healthcare system in the United States, use of electronic medical records data rather than patient self-report for determination of healthcare utilization, and prospective, detailed utilization and cost data. The study also has several limitations. First, insomnia is frequently treated but not often diagnosed [26]. We attempted to address this discrepancy by identifying insomnia through prescription fills for sedative-hypnotics; however, the indication for the prescribed medications could not be determined. Diagnosis of insomnia was based on ICD-9-CM and ICD-10-CM codes, yet the criteria used by providers for determining diagnosis is unclear. Therefore, the number of patients with insomnia in our study may over- or under-represent the true prevalence. Second, the use of ICD codes for the identification of COPD diagnosis could have led to misdiagnosis or underdiagnosis of COPD in our study. Additionally, the severity of COPD was unable to be reported and may be an important factor influencing healthcare use and costs. Unfortunately, availability of spirometry and Global Initiative for Chronic Obstructive Lung Disease data to confirm ICD diagnosis and severity of COPD was very limited in our study cohort due to variability in data collection, often which requires natural language processing of notes to extract relevant data. Prior research reported that sleep disturbance remained longitudinally associated with respiratory-related emergency utilization, even after controlling for forced expiratory volume in 1 s and COPD severity based on a validated survey [12]. Third, this study included veterans within the VA, thus studies within other healthcare systems are needed to confirm associations between insomnia and healthcare utilization and costs among patients with COPD. Finally, because utilization outcomes were extracted from VHA administrative data, we were unable to capture utilization that occurred outside of the VA. Furthermore, given that the study was focused on the VHA system, costs for healthcare delivered outside of the VHA system was not captured and thus the findings may not generalize to other healthcare delivery systems. Future studies should evaluate healthcare utilization across multiple healthcare systems or take into account provider visits that occurred outside on patients’ primary systems.

## Conclusion

This study suggests that insomnia is common among patients with COPD and is associated with increased COPD-related healthcare utilization, including prescription fills for steroids and/or antibiotics, outpatient visits, and hospitalizations. Proper management of COPD and other comorbid conditions may help to resolve insomnia symptoms; however, routine assessment of insomnia and initiation of insomnia treatment should be considered. Insomnia is often undiagnosed [26] and could be a potentially modifiable target for reducing the burden of COPD on patients and healthcare systems.

## Data Availability

Data are not public but may be available upon reasonable request to the corresponding author.

## List of abbreviations

BMI: body mass index
CBT-I: cognitive-behavioral therapy for insomnia
COPD: chronic obstructive pulmonary disease
FY: fiscal year
ED: emergency department
ICD-9-CM: International Classification of Diseases, Ninth Revision, Clinical Modification
ICD-10-CM: International Classification of Diseases, Tenth Revision, Clinical Modification
VA: Veteran Affairs
VHA: Veterans Health Administration
